# Beneath the Cure Lies the Risk: Carbimazole-Induced Angioedema in Graves' Disease

**DOI:** 10.7759/cureus.100860

**Published:** 2026-01-05

**Authors:** Judat Tasawoor, Mariya Manzoor, Abir Aijaz, Zagham Hammad, Hadiya Chisti, Ayesha Babar, Manzoor Wani, Abdul Bhat

**Affiliations:** 1 Medicine, Sub-District Hospital, Sopore, IND; 2 Acute Medicine, University Hospitals Bristol and Weston, Weston Supermare, GBR; 3 Acute and General Internal Medicine, University Hospitals Bristol and Weston, Weston Supermare, GBR; 4 Medicine, University Hospitals Bristol and Weston, Weston Supermare, GBR; 5 Acute Medicine, Weston General Hospital, University Hospitals Bristol and Weston, Weston Supermare, GBR

**Keywords:** carbimazole, drug-induced angioedema, graves´disease, skin eruptions, urticaria

## Abstract

Carbimazole is a widely used antithyroid medication for the treatment of hyperthyroidism and is generally well tolerated. Most adverse effects are mild and limited to cutaneous reactions. However, angioedema is a rare but potentially serious hypersensitivity response that may occur shortly after drug initiation.

We describe a patient who developed acute facial angioedema soon after starting carbimazole therapy. The patient presented with sudden periorbital and lip swelling accompanied by mild difficulty in swallowing, without any respiratory compromise. There was no history of allergies, autoimmune disease, or exposure to other new medications. Clinical examination revealed non-pitting edema of the eyelids and lips without associated urticaria. Given the close temporal relationship between carbimazole initiation and symptom onset, the drug was considered the most likely cause. Carbimazole was promptly discontinued, and treatment with antihistamines and corticosteroids resulted in complete resolution of symptoms within two days. No recurrence was observed following drug withdrawal, and the patient later underwent definitive treatment for hyperthyroidism.

This case adds to the limited literature on carbimazole-induced angioedema and highlights the importance of early recognition of rare hypersensitivity reactions to antithyroid drugs. Prompt drug discontinuation and appropriate management can prevent progression to life-threatening airway involvement. Increased awareness of this uncommon adverse effect may improve patient safety and clinical outcomes during antithyroid therapy.

## Introduction

Carbimazole, a prodrug that is converted to methimazole, is one of the most commonly prescribed antithyroid medications for the treatment of hyperthyroidism, particularly Graves’ disease. By inhibiting thyroid peroxidase, it suppresses thyroid hormone synthesis and provides effective biochemical and symptomatic control in most patients. Its convenient dosing and predictable clinical response have made it a first-line option in routine practice [1,2].

Although carbimazole is generally well tolerated, it is associated with a range of adverse effects. Mild reactions, such as gastrointestinal upset and cutaneous eruptions, are relatively common and usually manageable. More serious complications, including agranulocytosis, are rare but potentially life-threatening and typically occur within the first few months of therapy. Hypersensitivity reactions affect a small proportion of patients and most often present as pruritic maculopapular rashes or urticaria, which may necessitate drug withdrawal if persistent [3,4].

Angioedema is an exceptionally rare adverse reaction to carbimazole. It is characterized by sudden swelling of the deeper layers of the skin or mucosa, most commonly involving the lips, tongue, face, or eyelids. When the upper airway is affected, the condition can rapidly become life-threatening. Reported cases suggest that onset may occur within hours to days of drug exposure, making early recognition crucial. Because autoimmune thyroid disease itself may be associated with urticaria or angioedema, distinguishing drug-induced reactions from disease-related manifestations can be challenging [5,6].

Given the rarity of carbimazole-induced angioedema and the limited number of well-documented cases, reporting such presentations is important to improve clinical awareness. This case report aims to highlight the early clinical features, management, and outcome of carbimazole-induced angioedema and to emphasize the importance of prompt drug discontinuation to prevent airway compromise.

## Case presentation

**Figure 1:**
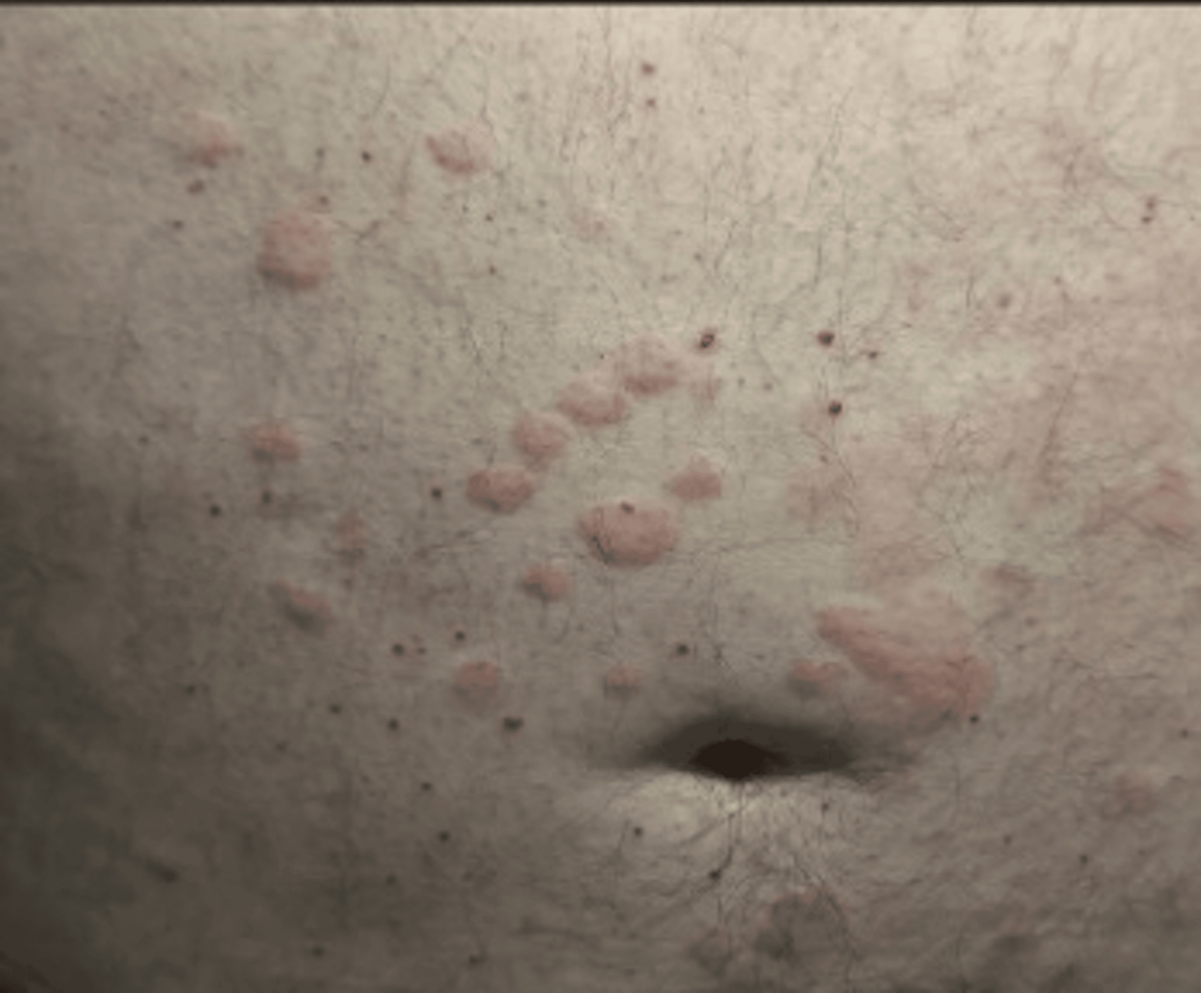
Uticarious rash with hives noted on abdomen

**Figure 2:**
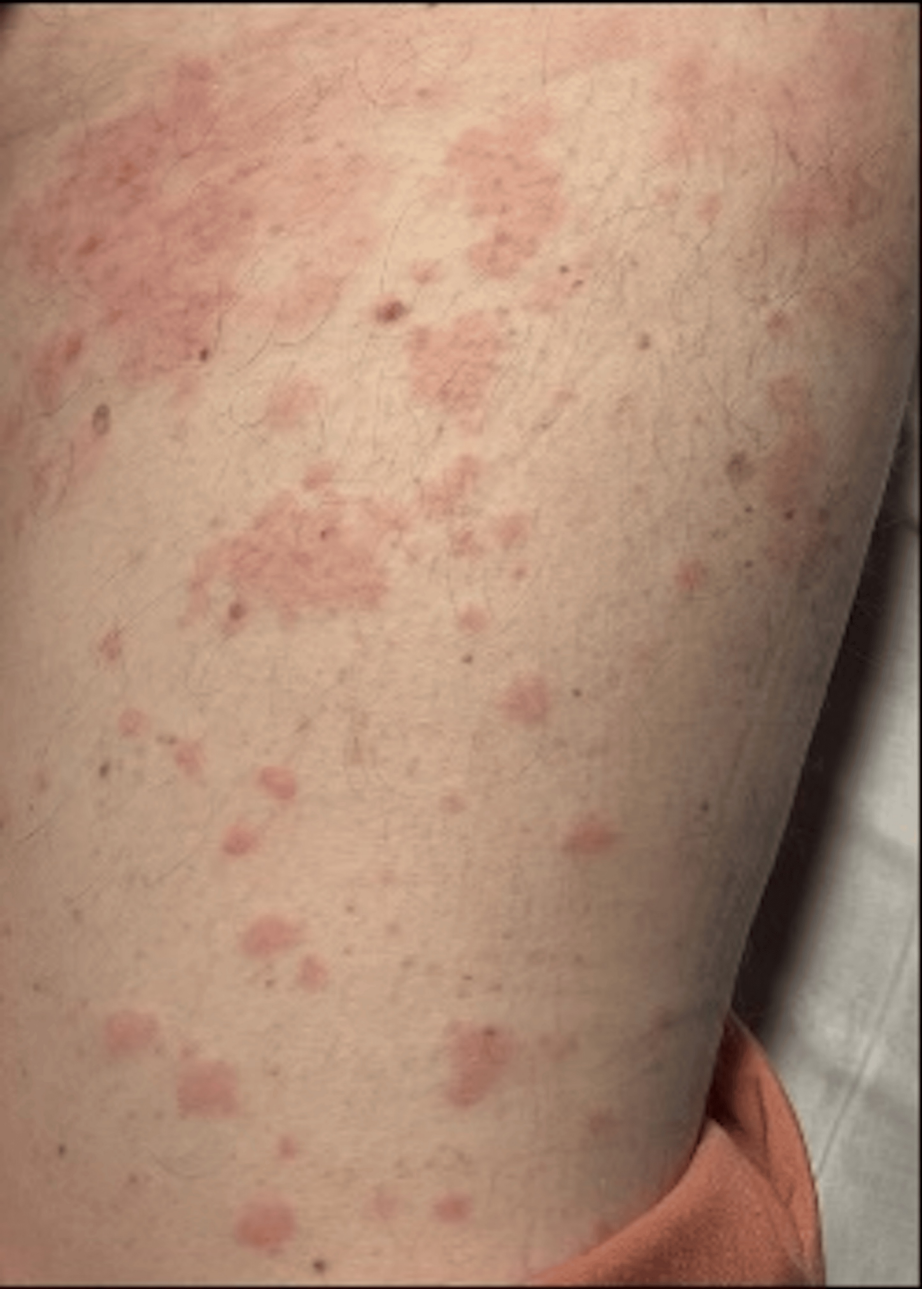
Rash on limbs

A 60-year-old man with newly diagnosed Graves’ disease was started on carbimazole 20 mg daily for thyrotoxicosis. Three days later, he presented to the emergency department with a sudden onset of lip and tongue swelling accompanied by widespread, itchy wheals across his trunk and upper limbs (Figures 1, 2).

He denied shortness of breath, wheezing, chest pain, recent dietary changes, insect bites, or exposure to any new medications other than atenolol, which had been prescribed earlier for symptomatic control of palpitations.

On examination, his vital parameters were stable: heart rate, 104 beats/min (irregularly irregular); blood pressure, 126/78 mmHg; oxygen saturation, 97% on room air; and respiratory rate, 16 breaths/min. There was visible swelling of the lips and anterior tongue, but no stridor or drooling. Urticarial lesions were noted over the torso and upper arms. Cardiovascular examination confirmed atrial fibrillation without signs of heart failure, and chest and abdominal examinations were unremarkable.

Laboratory tests demonstrated a suppressed thyroid-stimulating hormone (TSH) with elevated free T3 and T4 levels, consistent with thyrotoxicosis. Full blood count, renal, and liver function tests were within normal limits. There was no eosinophilia. Serum tryptase and complement C1-esterase inhibitor levels were normal, effectively excluding mast-cell activation and hereditary angioedema. Based on clinical findings and temporal association, a diagnosis of carbimazole-induced angioedema with urticaria was made.

Carbimazole was immediately discontinued. The patient received intravenous hydrocortisone (100 mg) and chlorpheniramine (10 mg) along with close airway observation in a monitored setting. His urticaria resolved within 72 hours, and the angioedema subsided markedly within 36 hours. No airway intervention was required. He was subsequently commenced on propylthiouracil (PTU) as an alternative antithyroid agent, which was well tolerated. Radioiodine ablation was later performed to achieve definitive control of his Graves’ disease. At six-month follow-up, he remained euthyroid and free from any hypersensitivity recurrence.

## Discussion

Carbimazole, via its active metabolite methimazole, is an effective and widely used agent for the medical management of Graves’ disease. While common adverse effects include gastrointestinal upset, arthralgia, and mild cutaneous reactions, angioedema remains an exceptionally rare manifestation of hypersensitivity. Published literature suggests that such reactions typically occur early after drug exposure, often within days to weeks of initiation, which is consistent with the clinical course observed in this patient [6]. The underlying mechanism of carbimazole-induced angioedema is not fully understood, but proposed pathways include IgE-mediated hypersensitivity as well as non-IgE-mediated mast cell activation. The absence of elevated tryptase levels in some reported cases, including ours, supports the possibility of idiosyncratic or non-immunologic mechanisms [7].

Only a limited number of carbimazole-associated angioedema cases have been documented, underscoring the rarity of this reaction. The reported presentations commonly involve facial, lip, or tongue swelling, with variable association with urticaria and generally favorable outcomes following prompt drug withdrawal and supportive therapy [8]. Similar to previously described cases, our patient experienced rapid symptom resolution after discontinuation of carbimazole, reinforcing the reversibility of this reaction when recognized early. In contrast, some reports describe progression to more severe anaphylactic reactions requiring airway intervention, highlighting the spectrum of severity and the importance of early identification [9].

Differentiating drug-induced angioedema from autoimmune-related urticaria or angioedema associated with Graves’ disease can be challenging. Autoimmune-mediated angioedema is typically chronic or recurrent, whereas drug-induced reactions tend to have an abrupt onset and resolve completely after cessation of the offending agent. In our case, the clear temporal relationship between carbimazole initiation and symptom onset, along with sustained resolution after withdrawal, supports a drug-related etiology rather than an autoimmune manifestation [10].

Management strategies reported in the literature consistently emphasize immediate discontinuation of carbimazole, administration of antihistamines and corticosteroids, and close airway monitoring [11]. This approach was effective in our patient who did not require advanced airway support. Although cross-reactivity between carbimazole and propylthiouracil has been described, several studies indicate that many patients tolerate PTU without recurrence of hypersensitivity, as seen in this case [5,7]. Nevertheless, definitive treatment with radioiodine ablation or surgery is often recommended to avoid repeated exposure to thionamides, particularly in older patients or those who experience severe reactions [9,10].

Overall, this case aligns with existing reports regarding timing, presentation, and response to treatment, while adding to the limited pool of documented carbimazole-induced angioedema cases. Increased reporting of such reactions is essential to better define risk factors, guide management decisions, and improve patient safety during antithyroid drug therapy.

## Conclusions

This case demonstrates that carbimazole can rarely precipitate acute angioedema with urticaria within days of treatment initiation. Sudden lip or tongue swelling in patients receiving carbimazole should prompt immediate drug discontinuation and close airway monitoring.

Early intervention led to rapid symptom resolution in this patient without airway compromise. Propylthiouracil was safely tolerated, and definitive treatment with radioiodine ablation prevented recurrence. Awareness of this rare reaction is essential to ensure timely management and patient safety during antithyroid therapy

## References

[1] Awosika AO (2023). Methimazole. StatPearls.

[2] Nakamura H, Miyauchi A, Miyawaki N, Imagawa J (2013). Analysis of 754 cases of antithyroid drug-induced agranulocytosis over 30 years in Japan. J Clin Endocrinol Metab.

[3] Wang Y, Li X, Yang Q (2019). Granulocyte-colony-stimulating factor effectively shortens recovery duration in anti-thyroid-drug-induced agranulocytosis: a systematic review and meta-analysis. Front Endocrinol (Lausanne).

[4] Angelis S, Trellopoulos A, Kondylis AK (2019). Multifocal osteomyelitis localization after pyomyositis in children: importance of timely response. Cureus.

[5] Yu W, Wu N, Li L, Wang J, OuYang H, Shen H (2020). Side effects of PTU and MMI in the treatment of hyperthyroidism: a systematic review and meta-analysis. Endocr Pract.

[6] Taylor PN, Vaidya B (2012). Side effects of anti-thyroid drugs and their impact on the choice of treatment for thyrotoxicosis in pregnancy. Eur Thyroid J.

[7] Cooper DS, Goldminz D, Levin AA, Ladenson PW, Daniels GH, Molitch ME, Ridgway EC (1983). Agranulocytosis associated with antithyroid drugs. Effects of patient age and drug dose. Ann Intern Med.

[8] García Gómez C, Navarro E, Alcázar V (2023). Therapeutic management and long-term outcome of hyperthyroidism in patients with antithyroid-induced agranulocytosis: a retrospective, multicenter study. J Clin Med.

[9] Ross DS, Burch HB, Cooper DS (2016). 2016 American Thyroid Association Guidelines for Diagnosis and Management of Hyperthyroidism and Other Causes of Thyrotoxicosis. Thyroid.

[10] Keyal NK, Thapa S, Yadav MK (2019). Carbimazole-induced anaphylactic shock: a case report. Indian J Crit Care Med.

[11] Garg D, Garcia GA, Praw SS (2021). A rare case of anaphylaxis to methimazole in a patient with Graves’ disease. J Endocr Soc.

